# Supplementary material for: Comparison of Hemodynamic Factors Predicting Prognosis in Heart Failure: A Systematic Review
Source: J Clin Med. 2019 Oct 22;8(10):1757. doi: 10.3390/jcm8101757 (PMC6832156; doi:10.3390/jcm8101757)
Supplement: Supplementary file 1 [file jcm-08-01757-s001.zip › jcm-607014-supplementary/Supplement 1 Search strategy.docx]

### **Supplement 1: Search strategy**

Pubmed search

Search 1 (25-01-2018) → results: 83

Heart Failure[Mesh] AND Prognosis[Mesh] AND Cardiac Output[Mesh] AND Pulmonary Wedge Pressure[Mesh]

Search 2 (25-01-2018) → results: 168

Heart Failure[Mesh] AND Prognosis[Mesh] AND Blood Pressure[Mesh] AND Pulmonary Wedge Pressure[Mesh]

Search 3(25-1-2018) → results: 381

Heart Failure[Mesh] AND Prognosis[Mesh] AND Blood Pressure[Mesh] AND Cardiac Output[Mesh]

Embase search

1. Heart failure or heart failure (txt)

2. Prognosis

3. 1+2 combined

4. cardiac output (txt)

5. 3+4 combined

6. pulmonary wedge pressure (txt)

7. 5+6 combined -> results 13

1.Heart failure OR heart failue (txt)

2. Prognosis

3. 1+2 combined

4. blood pressure (txt)

5. 3+4 combined

6. pulmonary wedge pressure (txt)

7. 5+6 combined -> results 28

1.Heart failure OR heart failure (txt)

2. Prognosis

3. 1+2 combined

4. cardiac output (txt)

5. 3+4 combined

6. blood pressure (txt)

7. 5+6 combined -> results 117
