# Supplementary material for: Comparison of Hemodynamic Factors Predicting Prognosis in Heart Failure: A Systematic Review
Source: J Clin Med. 2019 Oct 22;8(10):1757. doi: 10.3390/jcm8101757 (PMC6832156; doi:10.3390/jcm8101757)
Supplement: Supplementary file 1 [file jcm-08-01757-s001.zip › jcm-607014-supplementary/Supplement 2A- Complete Critical Appraisal methods & results .docx]

### **Supplement 2A: Complete Critical Appraisal methods & results**

**Methods**

Eight critical appraisal items included: 1. Adequate number of patients in HF-population (<200 or >200); 2. CO measurement: by Fick method (best method) versus thermodilution and unreported methods; 3. Outcome measurement valid and reliable: presence or absence of an adjudication committee/methods including external data validation; 4. Study attrition: follow-up done in $\geq$90% of patient population; 5. Age (or other important predictive variables, see below) was present in MV-analysis; 6. At least two other univariately significant variables present in UV-analysis; 7. Number of events (<50 or >50) and 8. Ratio of number of events per number variables tested <10 [45]. Our definition of “important predictive variables” for prognosis in HF were: age, renal function (e.g. blood urea nitrogen, GFR), sodium, ejection fraction, brain natriuretic peptide (N-Terminal Pro-brain natriuretic peptide) and severity of heart failure according to the New York Heart Association classification.

**Results**

Of the 20 articles that performed UV- or MV-analysis, CO was measured by thermodilution in 12 studies, and with Fick method in 1 study [28]; in the 7 remaining studies it was unreported (see Table 2).
