# Supplementary material for: Comparison of Hemodynamic Factors Predicting Prognosis in Heart Failure: A Systematic Review
Source: J Clin Med. 2019 Oct 22;8(10):1757. doi: 10.3390/jcm8101757 (PMC6832156; doi:10.3390/jcm8101757)
Supplement: Supplementary file 1 [file jcm-08-01757-s001.zip › jcm-607014-supplementary/Supplements 5A B and C Direct comparisons Figure results and table.docx]

### **Supplement 5A: Direct Comparisons Figure**

Cardiac Output

[24]–[26], [28] [16], [21], [24]–[28]

PCWP

SBP

[14], [18], [24]–[26], [28]–[30]

Overview of the number of studies with comparisons between hemodynamic variables in MV-analysis. References of the studies are given.

### **Supplement 5B: Direct Comparisons Methods and Results**

**Methods**

A fifth analysis was performed with a direct comparison between the hemodynamic variables, Age, VO2-max and NT-proBNP in MV-analysis, of which at least two were tested against each other. Therefore a separate table was made comparing how many times a variable remained in MV-analysis in the presence of another variable. In this way, the relevance of a singular variable was tested as ‘best’ hemodynamic variable or as ‘less relevant’ variable of the three hemodynamic variables.

**Results**

In Supplement 5C, two MV prognostic variables are tested against each other and the number of studies that show a prognostic significance are shown divided by the number of studies that studied the comparison in MV-analysis. Supplement 5A illustrates the comparisons that were made in which studies. For our study on MV-predictors (Supplement 4B (Data Analysis: MV-analysis)), all three hemodynamic variables were either univariately or multivariately tested in 12 of 18 included studies that finally performed a MV-analysis.

CI was not a significant predictor in any MV-analysis against another variable. There was no specific predictor against which PCWP did not retain significance in MV-analysis.

SBP remained significant in 1 study where PCWP lost significance [29], in 3 studies SBP did not remain significant against PCWP [24,25,28], 3 studies where both SBP and PCWP lost significance [14,26,30] and 1 study in which both PCWP and SBP remained significant [18].

Presence of confounding variables:

Age was present as UV-variable in 14 of 20 studies, and as MV-variable in only 4 of the 18 included studies with a MV-analysis [20,21,22,26].

VO2-max was present as UV-variable in 6 studies, and as MV-variable in 4 of 18 included studies with a MV-analysis [13], [14], [18], [25].

Natriuretic peptides was present as UV-variable in 4 studies, and as MV-variable in 4 of 18 included studies with a MV-analysis [15], [16], [18], [30].

### **Supplement 5C: Direct Comparisons Table in MV analyses**

|  |  | Variables (CI, PCWP, SBP) tested in MV analysis against: | | | |  |  |  |
| --- | --- | --- | --- | --- | --- | --- | --- | --- |
|  |  | **CI** | **PCWP** | **SBP** | **Age** | **VO2-max** | **Natriuretic peptides** | **Total** |
| **CI** | SIGN + | - | 0 of 7 | 0 of 4 | 0 of 2 | 0 of 1 | 0 of 1 | 0 of 15 |
|  | NS - | - | 7 of 7 | 4 of 4 | 2 of 2 | 1 of 1 | 1 of 1 |  |
| **PCWP** | SIGN + | 5 of 6 | - | 4 of 8 | 1 of 2 | 2 of 4 | 3 of 4 | 15 of 24 |
|  | NS - | 1 of 6 | - | 4 of 8 | 1 of 2 | 2 of 4 | 1 of 4 |  |
| **SBP** | SIGN + | 0 of 4 | 2 of 8 | - | 1 of 4 | 1 of 3 | 1 of 2 | 5 of 21 |
|  | NS - | 4 of 4 | 6 of 8 | - | 3 of 4 | 2 of 3 | 1 of 2 |  |
